# Rivaroxaban in patients with abdominal aortic aneurysm and high-sensitivity C-reactive protein elevation (BANBOO): study protocol for a randomized, controlled trial

**DOI:** 10.1186/s13063-023-07461-3

**Published:** 2023-06-19

**Authors:** Jingyuan Li, Sicong Ma, Xiu Jia, Yingzhen Bu, Tienan Zhou, Lei Zhang, Miaohan Qiu, Xiaozeng Wang

**Affiliations:** 1 College of Life Science and Biopharmaceutical, Shenyang Pharmaceutical University, 103 Wenhua Road, Shenhe District, Shenyang, 110016 Liaoning China; 2 Department of Cardiology, General Hospital of Northern Theatre Command, Liaoning 110016 Shenyang, China

**Keywords:** Abdominal aortic aneurysm, High-sensitivity C-reactive protein, Rivaroxaban; Randomized controlled trial, Protocol

## Abstract

**Background:**

Abdominal aortic aneurysm (AAA) is a fatal disease due to the tendency to rupture. The drug treatment for small AAA without surgical indications has been controversial. Previous studies showed that high-sensitivity C-reactive protein (hs-CRP) had become a potential biomarker of the disease, and the anti-inflammatory effect of rivaroxaban for AAA had been well established. Thus, we hypothesized that rivaroxaban could control the progression of AAA in patients with hs-CRP elevation.

**Methods:**

The study is a prospective, open-label, randomized, controlled clinical trial. Sixty subjects are recruited from the General Hospital of Northern Theatre Command of China. Subjects are randomly assigned (1:1) to the intervention arm (rivaroxaban) or control arm (aspirin). The primary efficacy outcome is the level of serum hs-CRP at 6 months. The secondary outcomes include imaging examination (the maximal diameter of AAA, the maximal thickness of mural thrombus, and the length of aneurysm), major adverse cardiovascular and cerebrovascular events (MACCE, including AAA transformation, non-fatal myocardial infarction, acute congestive heart failure, stent thrombosis, ischemia-driven target vessel revascularization, vascular amputation, stroke, cardiovascular death, and all-cause death), and other laboratory tests (troponin T, interleukin 6, D-dimer, and coagulation function).

**Discussion:**

The BANBOO trial tested the effect of rivaroxaban on the progression of AAA in patients with elevated Hs-CRP for the first time.

**Trial registration:**

ChiCTR2100051990, ClinicalTrials.gov, registered on 12 October 2021.

**Supplementary Information:**

The online version contains supplementary material available at 10.1186/s13063-023-07461-3.

## Introduction

### Background and rationale {6a}

Abdominal aortic aneurysm (AAA) is a chronic progressive disease that occurs mainly among males above 65 [1]. The incidences of AAA for males and females over 60 years were shown to be 5% and 1% [2]. Smoking and hypertension could promote the rupture risk of an aneurysm. Mortality could rise to 50–80% once rupture [3]. The drug treatment for “small” AAA, defined as the maximal aortic external diameter between 30 and 55 mm, has been controversial for individuals without surgical indications [4]. There are two main controversial points: whether the progression of AAA can be delayed or prevented and whether the fatal rupture incidence of AAA can be reduced by medical treatment.

A large number of evidence indicate that aortic wall inflammation is pivotal to the pathogenesis of AAA [5]. Regulating the expression of inflammation-related genes is a promising strategy for inhibiting the progression of AAA. The inflammatory cytokine high-sensitivity C-reactive protein (hs-CRP) has been corroborated as a potential biomarker of AAA [5]. Hs-CRP has been verified to predict cardiovascular risk and holds a positive correlation with the size of AAA [6]. The progression, expansion, and rupture of AAA could be diminished successfully by the exogenous anti-inflammatory drug [7–9].

Studies have shown that the average level of hs-CRP decreased significantly by rivaroxaban treatment from baseline to the endpoint of research [10, 11]. In addition, for the pro-inflammatory activity of factor Xa in arterial thrombosis, the direct inhibition of factor Xa by rivaroxaban may particularly benefit antithrombosis [12]. Rivaroxaban inhibits tissue factors, which induce platelet aggregation [13]. Studies showed that factor Xa inhibitor rivaroxaban exerted anti-inflammatory and anti-oxidative stress effects in human AAA lesion, suggesting a role of factor Xa underlying the pathogenesis of AAA [14, 15].

Many preclinical studies suggested that rivaroxaban may have a beneficial effect on AAA [15]. There have been extensive randomized controlled trials (RCTs) to evaluate the safety and efficacy of rivaroxaban in cardiovascular and cerebrovascular diseases (stroke prevention, venous protection, and vascular protection) [16]. For example, in the EINSTEIN study [17, 18], the risk of a recurrent event was significantly lower in the rivaroxaban group (10 to 20 mg) than in the aspirin group, without a significant increase in bleeding events [19]. In the ATLASACS-TIMI46 study [20], the rivaroxaban group was superior in terms of net clinical outcomes (death, myocardial, stroke, or TIMI hemorrhage).

New oral anticoagulant rivaroxaban has the advantages such as convenient usage, predictable pharmacokinetic characteristics, no requirement for regular monitoring, accurate anticoagulant effect, and low risk of bleeding, especially intracranial hemorrhage [21]. However, no specific medical therapy has been recommended (III, A), due to absence of evidence showing the slowed expansion rate of an AAA [22]. Moreover, blood pressure control, statins, and antiplatelet therapy should be considered in all patients with AAA (IIa, B) [22]. Therefore, we chose aspirin as a comparator of rivaroxaban in this trial.

**Fig. 1:**
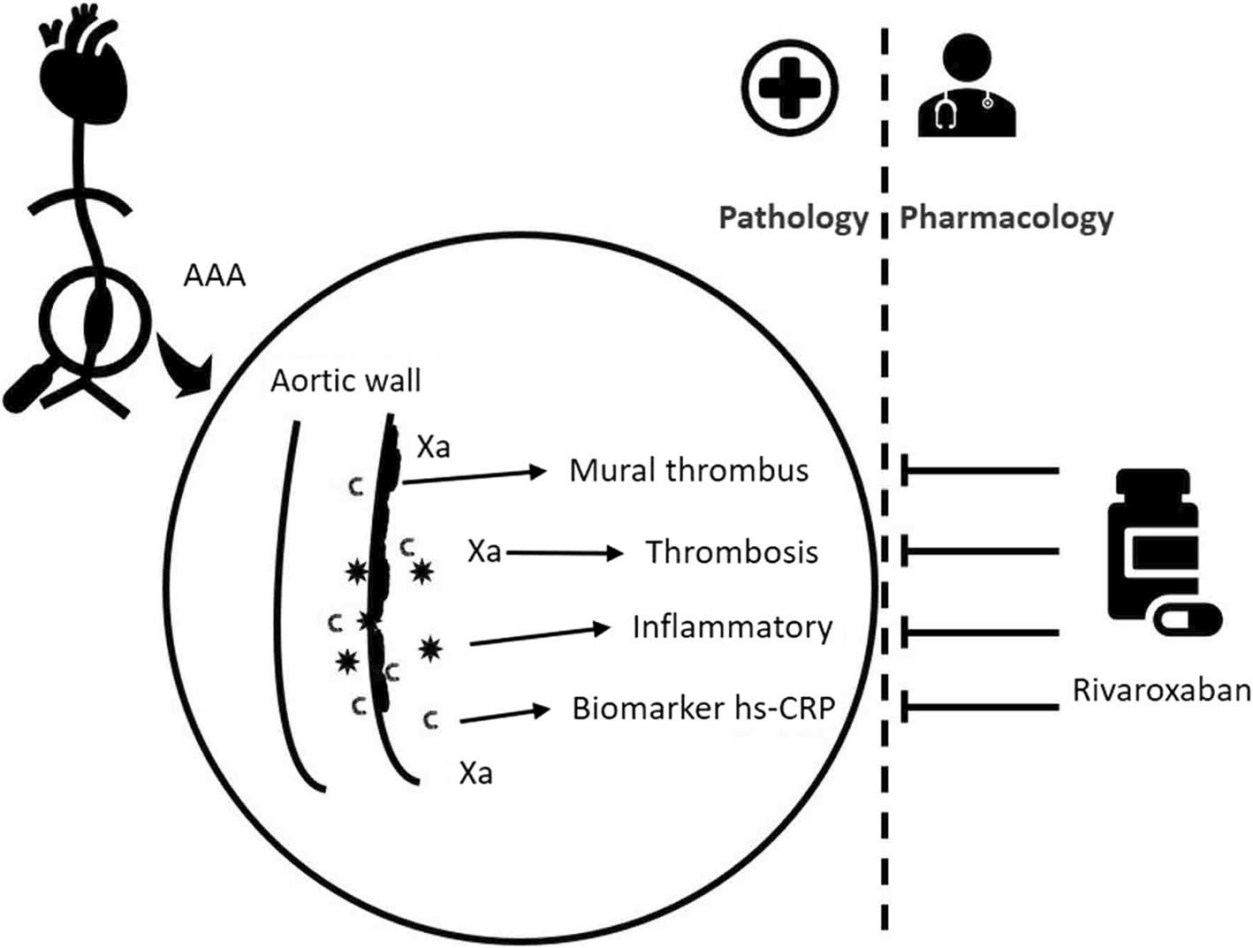
Study concept

We hypothesized that rivaroxaban had a good control effect on the progression of AAA in patients with elevated hs-CRP. The BANBOO study, a randomized controlled trial, will test this hypothesis, and its results will guide clinical practice (Fig. 1).

### Objectives {7}

The primary objective is to explore the anti-inflammatory efficacy of rivaroxaban in the treatment of AAA with hs-CRP elevation and to investigate the application value of rivaroxaban for AAA. The secondary objectives are to analyze the safety of rivaroxaban for AAA and the efficacy of rivaroxaban on antithrombosis and alleviating inflammatory lesions.

### Trial design {8}

**Fig. 2:**
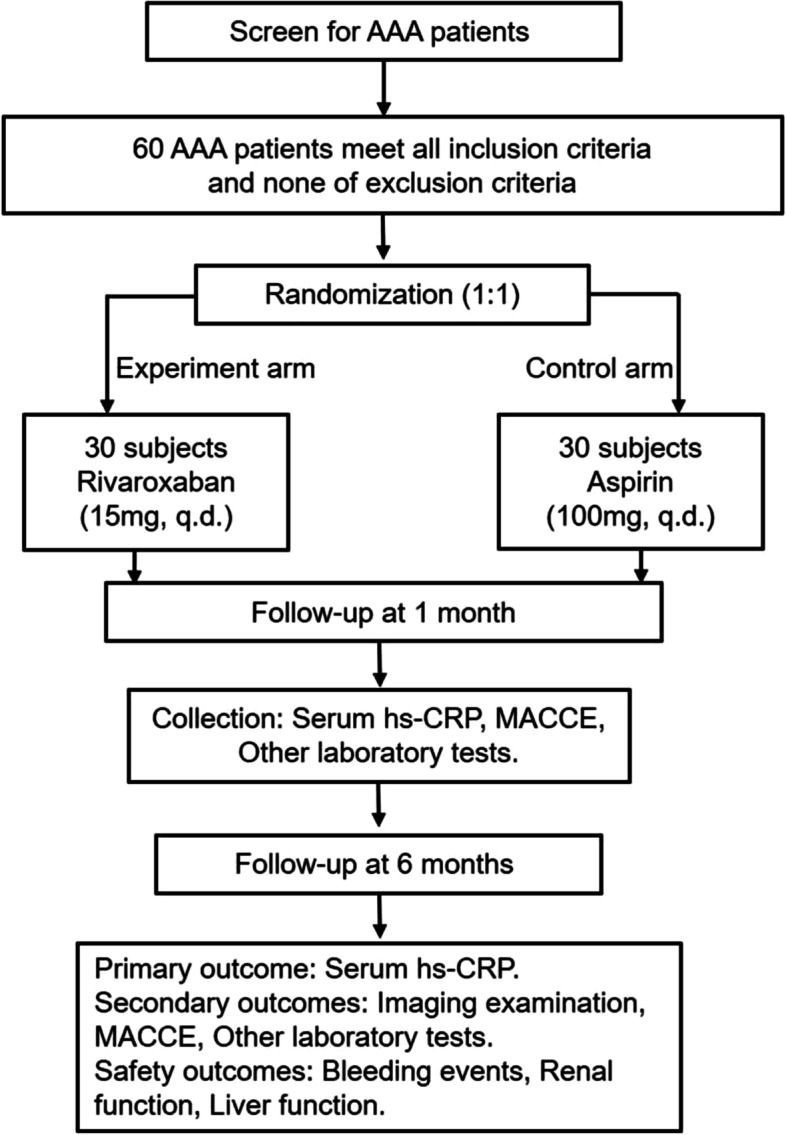
Research flowchart of the BANBOO trial

The BANBOO trial is a prospective, single-center, open-label, randomized, controlled clinical trial conducted in the General Hospital of Northern Theatre Command of China. The protocol was designed to adhere to the recommendations of the Standard Protocol Items for Randomized Trials statement (SPIRIT) [23–25]. The research flowchart of the BANBOO trial is detailed in Fig. 2.

## Methods: participants, interventions, and outcomes

### Study setting {9}

Initial baseline assessment includes contact information, physical examination, vital signs, demographical data, previous history, clinical characteristics, admission diagnosis, imaging examination, and laboratory test data. Once a standardized interview is performed, the data will be recorded in each subject’s case report form (CRF). Follow-up visits will occur at entry, 1 month, and 6 months after treatment initiation, and outcome data will be collected. Detailed collecting information on visits is listed in Table 1. An imaging subgroup is set up to analyze the effect of rivaroxaban on AAA morphology. All subjects screened, regardless of whether they are eligible for randomization, are provided with follow-up calls twice at 1 month and 6 months. Any interruption of treatments due to personal reasons is required to report within 24 h. Treatment should be discontinued immediately for emergent situations, including trauma or urgent invasive procedures, for the benefit of subjects.

**Table 1:** Collecting information on visits

Follow-up	Baseline (visit 1)	1 month (visit 2, ± 7 days)	6 months (visit 3, ± 7 days)
Inclusion/exclusion criteria	X		
Physical examination	X		
Vital signs	X		
ICF	X		
Contact information	X		
Demographic data	X		
Previous history	X		
Admitting diagnosis	X		
Subject guidance	X		
Drug therapy	X	X	X
hs-CRP	X	X	X
Imaging examination	X		X
MACCE		X	X
Other laboratory tests	X	X	X
Bleeding events (BARC)	X	X	X
Liver function	X	X	X
Renal function	X	X	X
Subject compliance		X	X

*ICF* inform consent form, *hs-CRP* high-sensitivity C-reactive protein, *MACCE* major adverse cardiovascular and cerebrovascular event, *BARC* Bleeding Academic Research Consortium

### Inclusion and exclusion criteria {10}

The BANBOO study is designed to enroll 60 AAA subjects with hs-CRP elevation. Subjects aged 18 to 85 years, 30–50 mm in diameter of the aneurysm, and serum hs-CRP ≥ 2 mg/L are eligible for enrollment. Each enrolled subject should provide written informed consent before randomization. Complete inclusion and exclusion criteria are listed in Table 2 and Table 3.

**Table 2:** Inclusion criteria

Inclusion criteria
1. 18–85 years old
2. AAA diagnosed by CTA; the maximal diameter of aneurysm is 30–50 mm
3. Serum hs-CRP ≥ 2 mg/L
4. Written informed consent

*AAA* abdominal aortic aneurysm, *CTA* computed tomography angioplasty, *hs-CRP* high-sensitivity C-reactive protein

**Table 3:** Exclusion criteria

Exclusion criteria
1. ACS (unstable angina and acute myocardial infarction)
2. Dual antiplatelet therapy for stable CHD less than 6 months after PCI or ACS less than 1 year after PCI
3. Acute congestive heart failure or left ventricular ejection fraction ≤ 40%
4. Suffered from infectious diseases within 2 months before screening and infection has not been controlled more than 1 month
5. Active hepatitis, the elevation of alanine aminotransferase (ALT) value > 5 × the upper limit of normal
6. Severe renal failure (CrCl < 30 ml/min)
7. Life expectancy is less than 1 year
8. Any situation may interfere with the research process, such as dementia, paralysis, alcoholism, etc
9. Pregnancy or women during the lactation period
10. Suffered from hereditary connective tissue disease, such as Marfan’s syndrome, etc
11. Known allergies or intolerance to aspirin or rivaroxaban
12. AAA tends to rupture or has ruptured, and abdominal pain aggravates
13. Major surgery within 1 month
14. Active stage of severe peptic ulcer or previous bleeding events (including retinal or vitreous hemorrhage, urinary tract hemorrhage, etc.) within 6 months
15. Have participated in other ongoing clinical studies
16. Refuse to provide a written informed consent
17. Other unsuitable conditions adjudicated by investigators

*ACS* acute coronary syndrome, *CHD* coronary heart disease, *PCI* percutaneous coronary intervention, *CrCl* creatinine clearance rate, CrCl = body weight(kg) × (140-age)/[0.818 × CREA(μmol/L)][serum creatinine (CREA) is tested by enzymatic method. Female: above data results × 0.85]

### Consent or assent {26a}

Tienan Zhou and Lei Zhang are responsible for obtaining written informed consent from participants who meet the inclusion criteria but not the exclusion criteria and are willing to provide written informed consent.

### Interventions

#### Choice of comparator {6b}

The study used aspirin as a control group, as recommended by the AAA guidelines. In the control arm, aspirin (100 mg q.d., Aspirin Enteric-coated Tablets, Bayer) is started at the time of randomization for 6 months. Subjects also receive standard background therapy with pitavastatin (2 mg daily, LiQingZhi, Japan Kowa Co., LTD), according to clinical guidelines.

#### Description of intervention {11a}

In the intervention arm, rivaroxaban (15 mg q.d., Xarelto, Bayer) is applied at the time of randomization for 6 months; the rivaroxaban dose will be reduced to 10 mg q.d. for the subjects aged over 75 years old. Subjects also receive standard background therapy with pitavastatin (2 mg daily, LiQingZhi, Japan Kowa Co., LTD).

#### Criteria for discontinuing or modifying allocated intervention {11b}

Possible reasons for withdrawal include (but not limited to) the following: subject’s voluntary withdrawal; the doctor will withdraw the subject according to clinical indications; subjects were lost to follow-up. Other conditions that may lead to treatment suspension will be evaluated and determined by investigators. If the protocol or ICF needs to be amended, the researchers are required to submit the amendment to the ethics committee for approval. Approved protocols or ICF amendments will be available to researchers prior to implementation. If an overdose occurs during the study period, the investigator should immediately notify the study leader and the principal investigator within 24 h but no later than the second day.

#### Strategies to improve adherence to interventions {11c}

Investigators and monitors will be trained in the research program, CRF, and regimen of research drugs. All training must be documented, including the revision of training materials, trained members, and training dates. The purpose of the training was to emphasize the importance of timely medication to the subjects. In addition, researchers will contact subjects regularly by telephone, solve the problems raised by patients in time, and increase the compliance of patients. All drugs used in the BANBOO trial should be recorded in the appropriate location in the CRF.

#### Relevant concomitant care permitted or prohibited during the trial (11d)

Medications are recommended according to current clinical guidelines. If the subjects need to use other drugs, the doctors should be informed.

#### Ancillary and post-trial care {30}

All patients screened, regardless of whether they are eligible for randomization, are documented in the patient database of the trial center and provided with regular follow-up calls or face-to-face visits even post-trial. At the end of the trial, the subject’s attending physician will determine the subject should continue the treatment regimen.

### Outcomes {12}

The primary efficacy outcome is the level of serum hs-CRP at 6 months. The secondary outcomes include imaging examination (the maximal diameter of AAA, the maximal thickness of mural thrombus, and the length of aneurysm), major adverse cardiovascular and cerebrovascular events (MACCE, including AAA transformation, non-fatal myocardial infarction, acute congestive heart failure, stent thrombosis, ischemia-driven target vessel revascularization, vascular amputation, stroke, cardiovascular death, and all-cause death), other laboratory tests (troponin T, interleukin 6, D-dimer, and coagulation function). Blood will be taken at entry and 1 and 6 months to assess changes in circulatory indexes. CTA will be performed at entry and 6 months, depending on local ethics guidelines. The safety outcomes include bleeding events defined according to the Bleeding Academic Research Consortium criteria (BARC), liver function (determined by alanine aminotransferase), and renal function (determined based on the creatinine clearance rate). Detailed endpoints are listed in Additional file 1: Appendix A, and detailed definitions of endpoints are shown in Additional file 1: Appendix B. The results of the BANBOO trial will be interpreted based on all endpoints.

### Participant timeline {13}

The study is expected to last 18 months, including a 12 months enrollment period and a 6 months follow-up period.

### Sample size calculation {14}

Power calculations are based on a superiority comparison of the primary endpoint. Assuming the level of serum hs-CRP in the aspirin arm (control) is 4.79 ± 3.2 mg/L at 6 months, and taking the drop-out rate is 10%, 30 patients in each arm (60 in total) are planned to be enrolled and randomly assigned in a 1:1 ratio, to provide 90% power to detect a 2.54 mg/L absolute value reduction in rivaroxaban arm (intervention) in comparison with that in aspirin group with a 2-sided type I error of 0.05.

### Recruitment {15}

Subjects are recruited via the Internet (http://www.chictr.org.cn/index.aspx) and public places (such as the hospital bulletin board). The purpose is to publicize the study so that suitable patients can be enrolled in time. The BANBOO trial began to recruit in October 2021.

## Assignment of interventions: allocation

### Sequence generation {16a}

The randomized numbers were generated via computer by statistical investigators (Miaohan Qiu and Sicong Ma). This process was blinded to those researchers responsible for the conduct of the trial.

### Concealment mechanism {16b}

The randomized numbers were placed in opaque envelopes. The subject who decides to withdraw from the study will no longer be admitted to enter, and the subject number will not be assigned again.

### Implementation {16c}

Asymptomatic AAA is screened by CTA to determine the diameter of aortic aneurysm (30–50 mm). The maximal orthogonal AAA diameter on CTA is a measurement of infrarenal aortic diameter perpendicular to the lumen to avoid overestimation of the tortuous aorta. And then, the serum hs-CRP tests are performed in subjects with an eligible diameter of aortic aneurysm. Values of serum hs-CRP will be recorded at entry, 1 month, and 6 months. Immuno-turbidity is used to detect serum hs-CRP concentration. Eligible subjects are randomly assigned to the intervention arm (rivaroxaban) or control arm (aspirin) in a 1:1 ratio. Tienan Zhou and Lei Zhang will enroll the participants to their allocation.

## Assignment of interventions: blinding

### Blinding (masking) {17a}

Statistical researchers will be blinded to the allocation.

## Data collection, management, and analysis

### Data collection plan {18a}

CRF is used for data collection. Data is entered into the database, and all data need to be entered by two investigators. All data will be uploaded and updated on the Clinical Trial Management Public Platform (http://www.medresman.org.cn/ login.aspx).

### Plans to promote participant retention and complete follow-up {18b}

The investigator kept in touch with the subjects by telephone or WeChat and sent the results of each examination to the subjects to increase their compliance.

### Data management {19}

The data monitor was served by clinical trial experts who were not involved in this study. The monitor will check the data obtained from the trial and evaluate the arms of treatment. Monitors ensure that the trial team is conducting the study according to the protocol. All missing data needs to be accounted for and recorded in CRF. Investigators are required to sign off after completing the CRF.

### Confidentiality {27}

To protect the privacy of patients, truthfully inform the subject of the storage, use, and confidentiality measures of the subject’s personal information and do not disclose the subject’s personal information to a third party without authorization. Subjects provided written informed consent to allow the trial monitor to obtain and copy information in their medical records related to their participation in the study. As part of informed consent, the investigator will allow the trial monitor or regulatory authority to confidentially review any records that identify subjects in this study. This information can be shared with regulatory authorities; however, the trial monitor guarantees that personal and private information of the subjects will not be published elsewhere.

### Biological specimens {33}

The total amount of blood collected per patient at each visit was 18 ml. Blood samples for clinical laboratory tests are obtained by standard techniques and tested at local centers. The subject’s number and visit number should be pre-labeled on each specimen tube, at which time the researcher will add the patient’s personal information to the label. Biological specimens should be exhausted or disposed after statistical analysis.

### Additional consent provision for collection and use of participant data and biological specimens {26b}

The collection for a blood sample is informed on the ICF, and it is ensured that the results of each blood sample examination should be sent to the subject.

## Statistical considerations

### Statistical methods for primary and secondary outcomes {20a}

The primary endpoint analysis will be performed according to the intention-to-treat principle. The main aim of this study is to investigate whether rivaroxaban is superior to aspirin in reducing the level of serum hs-CRP in patients with AAA and hs-CRP elevation at 6 months. The null hypothesis (H0) for this analysis is that the level of the primary endpoint in the intervention arm is the same as that of the control arm, namely P0 = P1. The alternative hypothesis (H1) is that the levels of the primary endpoint in the two arms is not equal, namely P0 ≠ P1, and the superiority test is conducted at the 2-sided significance level of 0.05.

Continuous variables are presented as the mean ± standard deviation or the median with interquartile range and compared between two arms via *T*-test or Mann–Whitney *U* test. Categorical variables are presented as counts and percentages and compared between two arms via *χ*^2^ test. Kaplan–Meier curves and Log-rank tests are used to describe the cumulative incidences of MACCE and bleeding events. The differences in the risk of AAA between the two arms are estimated using a Cox proportional hazards model. A two-sided *P* < 0.05 is considered to be statistically significant. All statistical analyses are performed using the SAS (Version 9.3) software.

### Interim analyses {21b}

No formal interim analysis was planned. The monitors will be responsible for recommending that the investigators update the trial design or stop the trial early on the basis of clear evidence of harm or effectiveness before the plan is completed.

### Methods for population and missing data analysis {20c}

A per-protocol analysis is planned focused on subjects who regularly took the treatment drugs every day or nearly every day. Missing values will be imputed with the last available data or the mean value for that group.

## Oversite and monitoring {21a}

During the trial, the trial monitor will monitor the trial in accordance with the pre-specified monitoring plan. The trial monitor will review the accuracy and completeness of the study data at appropriate times and ensure compliance with the clinical study protocol. The study monitor may request all documents from the investigator as well as required records, including the medical records (office, clinic, or hospital) of the subjects in this study. Original documentation must be provided to confirm proper informed consent procedures, adherence to protocol procedures, proper reporting and follow-up of adverse events, the accuracy of data collected on case report forms, and study drug information.

## Adverse events or harms {22}

All adverse events (AEs) related to cardio-cerebrovascular, bleeding, liver, and renal damage were collected from the time subjects signed informed consent to the end of the study. The doctor should adopt the best treatment immediately after discovery and report to the ethics committee within 2 days of the occurrence. Based on study-eligible criteria, pregnant or would-be pregnant women are excluded from the study population. If unplanned pregnancy occurs during the study period, all pregnancies should be reported to the study leader and principal investigator; pregnant women should immediately discontinue research drugs and drop out of the study.

## Frequency and plans for auditing trial conduct {23}

The investigator agreed to give the auditor access to all relevant documents for review and support of the BANBOO trial. At the end of the trial, a copy of the audit certificate will be included in the final report.

## Communicating important protocol amendments to relevant parties {25}

It is necessary to inform the changes to all investigators. Important protocol modifications will be notified to the ethics committee and will be modified after being approved by the ethics committee. If a protocol modification affects the treatment recommendations that subjects should follow or may alter their perception of the risks or otherwise of the trial, this may lead to the requirement that investigators re-obtain informed consent.

## Dissemination policy

### Patient and public involvement {31a}

Before randomization, a training scheme for the eligible subjects and their relatives is initiated by professional investigators. The training covers the purpose and process of the BANBOO trial, possible benefits and potential risks, medication guidance, symptoms of AEs, the necessity to contact doctors actively, rights and responsibilities of subjects, and methods of disseminating the trial to other AAA patients. Researchers will answer the relevant questions from the subjects. Social media, such as WeChat, facilitate communication and connection between researchers and patients.

### Reproducible research {31c}

Findings will be disseminated via peer-reviewed journals and conferences. The BANBOO trial was registered and recruited on the Chinese Clinical Trial Registry (http://www.chictr. org.cn/index.aspx).

## Ethics statement {24}

All subjects must be fully informed of the trial process and provide written informed consent (Additional file 1: Appendix C). The trial is being conducted in accordance with the Declaration of Helsinki of the World Medical Association. The BANBOO trial has been approved by the Medical Ethics Committee of the General Hospital of Northern Theatre Command of China [Ethics Approval Number: Y(2021)090, Additional file 1: Appendix D]. Any amendments of protocol must be approved by ethics committee.

Researchers, ethics committees, monitors, and drug regulators will be allowed access to medical records and results. To protect patient privacy, subjects’ personal information shall not be disclosed in any public report of this study results or to a third party without authorization.

## Discussion

Life-threatening AAA requires monitoring or treatment depending on the size of the aortic aneurysm and/or symptoms. The current research on conservative treatment of AAA involves several aspects, such as lifestyle change, exercise therapy, medications, etc. Quit smoking is critical to current smokers; regular aerobic exercise does not reduce AAA growth and rupture [26]. There is an urgent need for drugs to control aneurysm growth or rupture, but the results of RCTs have been disappointing. Several preclinical studies on statins suggested potential benefits for AAA, but the results have not been verified by RCT [27]. Regarding antiplatelet medication, ticagrelor showed no difference in growth rates of AAA compared with placebo in RCT [28]. Propranolol has not been proved to be useful to reduce the growth rate of the aneurysm but increases side effects in the treatment arm [29]. There was no difference in growth rates among the perindopril, placebo, and placebo plus amlodipine group in a randomized trial, even though a decrease of blood pressure was detected in both perindopril and amlodipine group [30]. RCTs of macrolides in the treatment of AAA have yielded conflicting results, and more further studies are required [31–33].

Lacking effective drugs to prevent the progression of AAA has plagued researchers in this field [34]. Researchers continue to explore possible drugs to control AAA, and we are looking forward to exciting news from pending clinical studies (NCT04224051, NCT04723888, NCT02225756). Current guidelines, such as the National Institute for Health and Care Excellence (NICE) 2020 guideline on AAA: diagnosis and management of European Society for Vascular Surgery (ESVS) 2019 Clinical Practice Guidelines on the Management of Abdominal Aorto-iliac Artery Aneurysms [22, 35], are limited in recommendations for conservative medical treatment for lack of solid evidence. Therefore, more data are needed to support the conventional treatment of AAA.

There has been increasing evidence that aortic wall inflammation is the primary pathogenesis of AAA [5]. Studies showed that elevated hs-CRP positively correlates with AAA diameter size [36–38]. The concept that “lower is better” for hs-CRP influenced current guidelines [39, 40]. A study of 75 male patients and a study of 435 patients showed the serum hs-CRP as a marker of progression and expansion of AAA [41, 42]. One research suggested that hs-CRP is a valuable biomarker to assess the evolution of calcification [43]. Wang Y et al. evaluated the correlation between hs-CRP and AAA through systematic review including prospective studies, retrospective studies, and cohort studies. The results suggested that hs-CRP may possibly be used as a diagnostic biomarker in AAA patients with a medium to small aortic diameter but not in AAA patients with a large aortic diameter [44]. Hs-CRP could be a potential biomarker of AAA and a promising target to control the progression of AAA [8].

Studies showed a significant decrease in mean hs-CRP levels from baseline to the end of rivaroxaban treatment [10, 11]. The role of factor Xa in arterial thrombosis is considered due to its pro-inflammatory activity. The anti-inflammatory effects of rivaroxaban, a direct factor Xa inhibitor, may be particularly beneficial in inhibiting arterial thrombosis [12]. Rivaroxaban indirectly inhibits tissue factor-induced platelet aggregation by inhibiting thrombin production [13]. We hypothesize that rivaroxaban is a promising treatment strategy for small AAA with hs-CRP elevation. However, no clinical study of rivaroxaban in this field has been reported to date [45].

This study is a prospective, single-center, open-label, randomized controlled trial with a total sample size of 60 cases based on statistical calculation and the admission volume of patients with AAA in the research center. The study is blinded to the statistical investigator. The BANBOO trial is the first research to assess the efficacy and safety of rivaroxaban compared with aspirin in AAA patients with hs-CRP elevation.

## Current status

The BANBOO trial was initiated to recruit patients in October 2021. The estimated duration period of the BANBOO trial is 18 months. The study is ongoing.

All data of the BANBOO trial will be uploaded and updated on the Clinical Trial Management Public Platform (http://www.medresman.org.cn/login.aspx). All data will be reserved and analyzed in the database of the contract research organization.

## Supplementary Information

**Additional file1: Appendix A.** Detailed endpoints of the BANBOO trial.** Appendix B.** Endpoints’ definitions of the BANBOO trial.** Appendix C.** Patient consent for publication. {32}** Appendix D.** Ethics approval document.** Appendix E.** Funding document.

## Data Availability

All data of the BANBOO trial will be uploaded and updated on the Clinical Trial Management Public Platform (http://www.medresman.org.cn/login.aspx). All data will be reserved and analyzed in the database of the contract research organization.

## Abbreviations

AAA: Abdominal aortic aneurysm
AE: Adverse event
BARC: Bleeding Academic Research Consortium criteria
CRF: Case report form
CTA: Computed tomography angiography
ESVS: European Society for Vascular Surgery
hs-CRP: High-sensitivity C-reactive protein
ICF: Informed consent form
MACCE: Major adverse cardiovascular and cerebrovascular event
NICE: National Institute for Health and Care Excellence
RCT: Randomized controlled trial
SPIRIT: Standard Protocol Items for Randomized Trials statement
