# Supplementary material for: Rivaroxaban in patients with abdominal aortic aneurysm and high-sensitivity C-reactive protein elevation (BANBOO): study protocol for a randomized, controlled trial
Source: Trials. 2023 Jun 19;24:419. doi: 10.1186/s13063-023-07461-3 (PMC10280923; doi:10.1186/s13063-023-07461-3)
Supplement: Supplementary file 1 — Additional file1: Appendix A. Detailed endpoints of the BANBOO trial. Appendix B. Endpoints’ definitions of the BANBOO trial. Appendix C. Patient consent for publication. {32} Appendix D. Ethics approval document. Appendix E. Funding document. [file 13063_2023_7461_MOESM1_ESM.pdf]

Appendix A. Detailed endpoints of the BANBOO trial.

Primary outcome	
The level of serum hs-CRP at 6 months	
Secondary outcomes	
Imaging examination	
The maximal diameter of AAA	
The maximal thickness of mural thrombus	
The length of aneurysm	
MACCE	
AAA transformation	
Non-fatal myocardial infarction	
Acute congestive heart failure	
Stent thrombosis	
Target vessel revascularization	
Vascular amputation	
Stroke	
Cardiovascular death	
All-cause death	
Other laboratory tests	
Troponin T	
Interleukin 6	
D-dimer	
Coagulation function	
Safety outcomes	
Bleeding events	
Renal function	
Liver function	

hs-CRP, high-sensitivity C-reactive protein; AAA, abdominal aortic aneurysm; MACCE, Major adverse cardiovascular and cerebrovascular event.

Appendix B. Endpoints' definitions of the BANBOO trial.

1. The level of serum hs-CRP at 6 months

Three value of serum hs-CRP will be recorded at entry, 1month and 6 months in the BANBOO trial. Immuno-turbidity is used to detect serum hs-CRP concentration, measured in mg/L.

2. Imaging examination

The maximal orthogonal AAA diameter on computerized tomography angiography (CTA) is a measurement of infrarenal aortic diameter perpendicular to the lumen, devised to avoid over-estimation of tortuous AAAs, the largest orthogonal diameter is taken to be the maximal diameter of AAA. [Morris et al. TEDY. Trials (2015) 16:274. doi: 10.1186/s13063-015-0793-z]

The maximal thickness of mural thrombus is measured in the whole region of abdominal aorta (upper diaphragm, lower common iliac artery), not restricted at the site of maximal diameter.

The length of aneurysm is measured between the sites widen compared with the normal abdominal aorta, take care to correct tortuous part of AAAs by software (GE AW4.4 post-processing workstation).

3. Major adverse cardiovascular and cerebrovascular event (MACCE)

3.1 AAA transformation

AAA transformation is defined as AAAs develop into aortic dissection, penetrating ulcer, intramural hematoma, pseudoaneurysm and intraperitoneal hyperemia.

3.2 Myocardial infarction (MI) [Fourth Universal Definition of Myocardial Infarction (2018). Circulation. 2018 Nov 13;138(20):e618-e651. doi: 10.1161/CIR.0000000000000617.]

Myocardial infarction is defined according to the fourth Universal Definition of Myocardial Infarction.

Type 1 MI: spontaneous MI related to ischemia due to a primary coronary event such as plaque erosion and/or rupture, fissuring or dissection.

Type 2 MI: MI secondary to ischemia due to either increased oxygen demand or decreased supply.

Type 3 MI: death occurring before blood samples could be obtained, or at a time before the appearance of cardiac biomarkers in the blood.

Type 4a MI: MI associated with PCI.

Type 4b MI: MI associated with stent thrombosis.

Type 4c MI: MI associated with stent restenosis.

Type 5 MI: MI associated with Coronary Artery Bypass Graft(CABG).

3.3 Congestive heart failure [ESC Scientific Document Group. 2021 ESC Guidelines for the diagnosis and treatment of acute and chronic heart failure. Eur Heart J. 2021 Sep 21;42(36):3599-3726. doi: 10.1093/eurheartj/ehab368.]

Congestive heart failure (CHF) is a clinical syndrome with symptoms and/or signs caused by a structural and/or functional cardiac abnormality and corroborated by elevated natriuretic peptide levels and/or objective evidence of pulmonary or systemic congestion.

CHF has been divided into distinct phenotypes based on the measurement of left ventricular ejection fraction (LVEF).

Reduced LVEF is defined as ≤40%, i.e. those with a significant reduction in left ventricular (LV) systolic function. This is designated as HFrEF.

Patients with a LVEF between 41% and 49% have mildly reduced LV systolic function, i.e. HFmrEF. Retrospective analyses from RCTs in HFrEF or CHF with preserved ejection fraction (HFpEF) that have included patients with ejection fractions in the 40-50% range suggest that they may benefit from similar therapies to those with LVEF≤40%.This supports the renaming of HFmrEF from ‘heart failure with mid-range ejection fraction’ to ‘heart failure with mildly reduced ejection fraction’.

Those with symptoms and signs of CHF, with evidence of structural and/or functional cardiac abnormalities and/or raised natriuretic peptides, and with an LVEF≥50%, have HFpEF.

3.4 Stent thrombosis

Stent thrombosis is defined according to the definite or probable criteria of the Academic Research Consortium (Circulation 2007;115:2344-51).

3.4.1. Definite stent thrombosis

Presence of an ACS with angiographic or autopsy evidence of thrombus or occlusion.

3.4.2. Probable stent thrombosis

Unexplained death within 30 days after the procedure, or acute myocardial infarction involving the target-vessel territory without angiographic confirmation.

3.4.3. Possible stent thrombosis

All unexplained death occurring at least 30 days after the procedure.

Stent thrombosis classification by time frame：

1) Acute stent thrombosis

Occurring within 24 hours after the index PCI.

2) Subacute stent thrombosis

Occurring between 24 hours and 30 days after the index PCI.

3) Late stent thrombosis

Occurring between 31 and 360 days after the index PCI.

4) Very late stent thrombosis

Occurring later than 360 days after the index PCI.

3.5 Ischemia-driven Target Vessel Revascularization (ID-TVR)

Ischemia-driven target vessel revascularization is defined as repeat PCI or bypass surgery of the target lesion(s) and any additional lesions in the main epicardial coronary artery or branches containing the target lesion, with one or more of the following conditions:

1) Patient had ischemic symptoms and ECG-changes referable to the target lesion.

2) Diameter stenosis≥50% at follow-up angiography and a positive functional study corresponding to the area served by the target vessel.

3) Diameter stenosis<50% at follow-up angiography but a markedly positive functional study or ECG-modification corresponding to the territory supplied by target vessel.

4) Diameter stenosis≥70% at follow-up angiography in absence of documented clinical or functional ischemia.

3.6 Vascular amputation

Amputation due to limb necrosis, peripheral arteriosclerotic obliterans and thrombo-angiitis obliterans.

3.7 Stroke

Neurological dysfunction caused by acute focal lesions of the central nervous system due to vascular etiology, including hemorrhagic stroke and ischemic stroke. Patients present as sudden onset of vertigo, numbness, aphasia, dysarthria or central neurologic deficit secondary to vascular lesions of the brain such as hemorrhage, embolism, thrombosis, or rupturing aneurysm.

3.8 Cardiovascular death

Cardiovascular death is defined as death due to myocardial infarction, cardiac perforation or tamponade, arrhythmia, stroke during follow-up period, death due to a complication of the cardiovascular drugs allocated to subjects, and any death in which a cardiac cause cannot be excluded.

3.9 All-cause death

Deaths that are not caused by definite non-cardiac factors are deemed as cardiac deaths. Specifically, any unexpected deaths in subject are deemed as cardiac deaths, even if they also have potential fatal non-cardiac diseases (for example, cancer and infection).

4. Bleeding events

BANBOO trial is defined as Bleeding Academic Research Consortium (BARC) criteria. [Mehran R et al. Circulation. 2011 Jun 14;123(23):2736-47. doi: 10.1161/CIRCULATIONAHA.110.009449.]

Type 0: no evidence of bleeding.

Type 1: bleeding that is not actionable and does not cause the patient to seek unscheduled performance of studies, hospitalization, or treatment by a healthcare professional.

Type 2: any clinically overt sign of hemorrhage (eg, more bleeding than would be expected for a clinical circumstance, including bleeding found by imaging alone) that is actionable but does not meet criteria for type 3, type 4, or type 5 BARC bleeding. The bleeding must meet at least one of the following criteria: First, it requires intervention, defined as a health care professional guided medical treatment or percutaneous intervention to stop or treat bleeding, including temporarily or permanently discontinuing a medication or study drug. Second, the bleeding leads to hospitalization or an increased level of care, defined as leading to or prolonging hospitalization or transfer to a hospital unit capable of providing a higher level of care. Or third, the bleeding prompts evaluation, defined as leading to an unscheduled visit to a healthcare professional resulting in diagnostic testing.

Type 3a: Any transfusion with overt bleeding; Overt bleeding plus hemoglobin drop ≥3 to <5 g/dL.

Type 3b: Overt bleeding plus hemoglobin drop ≥5 g/dL; Cardiac tamponade; Bleeding requiring surgical intervention for control (excluding dental/nasal/skin/hemorrhoid); Bleeding requiring intravenous vasoactive drugs.

Type 3c: Intracranial hemorrhage (does not include microbleeds or hemorrhagic transformation; does include intraspinal); subcategories confirmed by autopsy, imaging, or lumbar puncture; Intraocular bleed compromising vision.

Type 4: CABG related bleeding; Perioperative intracranial bleeding within 48 hours; Reoperation after closure of sternotomy for the purpose of controlling bleeding; Transfusion of ≥5 U whole blood or packed red blood cells within a 48-hour period; Chest tube output ≥2 L within a 24-hour period.

Type 5: Fatal bleeding.

Appendix C. Patient consent for publication.

Informed consent

(Version 1.1)

Title: A randomized, controlled study of rivaroxaban in patients with abdominal aortic aneurysm and high-sensitivity C-reactive protein elevation (BANBOO)

Center: General Hospital of Northern Theatre Command, China

1. Research aim and profile

According your characteristics, you have been confirmed or suspected as being diagnosed with an abdominal aortic aneurysm (AAA). AAA is a chronic progressive disease that occurs mostly among male above 65 years old. The incidences of AAA for male and female aged over 60 years were shown to 5% and 1%.

Invasive surgery or interventional therapy is not recommended to the "small" AAA (defined as the maximal aortic external diameter between 30 and 55 millimeters) without surgical indications for the benefit of surgery can't outweigh the rupture risk. Closely monitoring the growth rate of the AAA is recommended, surgical or interventional treatment is required as soon as possible if the diameter exceeds 55 mm, or the growth rate is too rapid.

A large number of evidences indicate that rivaroxaban could diminish high-sensitivity C-reactive protein (hs-CRP) successfully to anti-inflammatory and prevent cardiovascular and cerebrovascular disease. So we invite you to participate in the BANBOO trial: A randomized, controlled study of rivaroxaban in patients with AAA and hs-CRP elevation. This is a clinical research study involving professional medical researchers. The study protocol has been approved by the Ethics Committee of General Hospital of Northern Theatre Command.

Before you decide whether to participate in the study, please read the following as carefully as possible. It can help you understand the study, why the study is being conducted, the process and duration of the study, the possible benefits and risks if you participate in the study. If you prefer, you can also discuss it with your relatives and friends, or ask your doctor for an explanation to help you make a decision. However, we cannot guarantee that rivaroxaban will work for you totally for

individual differences. If rivaroxaban does not work for your condition, you can ask your doctor about possible alternative treatments.

2. Protocols

This study was conducted in patients with AAA (aneurysm diameter of 30-50 mm) and hs-CRP elevation. The efficacy and safety of rivaroxaban in the treatment of AAA were investigated by the reduction degree of hs-CRP. Compared with aspirin group, the effect of rivaroxaban on preventing thrombosis and delaying disease evolution of AAA will be analyzed. This study was a prospective, single-center, open-label, randomized, controlled clinical trial.

2.1 Eligibility

Suitable for participants in this study:

1) 18-85 years old;

2) AAA diagnosed by CTA, the diameter of the aneurysm is 30-50mm;

3) Serum hs-CRP≥2mg/L;

4) Written Informed consent .

Unsuitable for participants in this study:

1) ACS (unstable angina and acute myocardial infarction);

2) Dual antiplatelet therapy for stable CHD less than 6 months after PCI or for ACS less than 1 year after PCI;

3) Acute congestive heart failure or left ventricular ejection fraction≤40%;

4) Suffer from infectious diseases within 2 months before screening and infection has not been controlled more than 1 month;

5) Active hepatitis , the elevation of alanine aminotransferase (ALT) values>5×the upper limit of normal;

6) Severe renal failure (CrCl<30ml/min);

7) Life expectancy is less than 1 year;

8) Any situation may interfere with the research process, such as dementia, paralysis, alcoholism etc.;

9) Pregnancy or women during suckling period;

10) Suffered from hereditary connective tissue disease, such as Marfan’s syndrome etc.;

11) Known allergies or intolerance to aspirin and rivaroxaban;

12) AAA tends to rupture or has ruptured, abdominal pain aggravates;

13) Major surgery within 1 month;

14) Active stage of severe peptic ulcer or previous bleeding events (including retinal or vitreous hemorrhage, urinary track hemorrhage etc.) within 6 months;

15) Have participated in other ongoing clinical studies;

16) Refuse to write an informed consent;

17) Other conditions unsuitable adjudicated by investigators.

2.2 Therapy procedure

If you meet the randomization criteria, you will be randomly assigned to either intervention (rivaroxaban) or control (aspirin) group. And then, if you are assigned to the intervention group, you will be treated with rivaroxaban tablets (XARELTO®, Bayer, 15mg once daily, 10mg for patients over 75 years of age); If you are assigned to the control group, you will be treated with aspirin enteric-coated tablets (BAYASPIRIN®, Bayer, 100mg once daily); Regardless of which group was assigned, pitavastatin calcium tablets (LiQingZhi®, Kowa Company Ltd., 2mg once daily) are administered.

All enrolled patients could complete 3 visits: the doctor will learn about your medication status, adverse events and relevant laboratory test items (include liver function, kidney function, hs-CRP, interleukin-6, coagulation, etc.) at 1 month and 6 months, all laboratory tests were routine tests of AAA. Imaging examination (CTA) at 6 months will be added as final visit.

2.3 Study time and number of patients

A total of 60 would be enrolled in the study. The approximate time span is 18 months.

2.4 Limitations and responsibilities of subjects

2.4.1 Before you are enrolled in this study, your medical history will be asked and recorded, and CTA examination and relevant laboratory tests will be performed to confirm the diagnosis of AAA. If your doctor determines that you are suitable to participate in the study according to the inclusion and exclusion criteria, you can voluntarily decide to participate in and sign the

informed consent or not. If you do not wish to participate in the study, your doctor will respect your choice and follow the local treatment guidelines of AAA.

2.4.2 During the follow-up period, the doctor will inform you of the time of face-to-face follow-up via phone/Wechat. Your follow-up is very important, because the doctor will judge the treatment effect and adverse events of drugs you have received. You should not use other antithrombotic drugs during the study period. If you need extra treatment, please contact your doctor in advance for more reasonable advices.

3. Side effects, risks and discomfort

3.1 Side effects and risks

Participants in both rivaroxaban and aspirin groups were at risk for adverse events, but this risk was not significant in many of the preliminary clinical trials. Both treatments are prone to bleeding. The risk of bleeding was considered when you were enrolled in the study, and your risk of bleeding is very low. There may be a risk of gastrointestinal bleeding in the aspirin group. Based on current evidence and guidelines, this increased risk is uncertain and can be prevented and controlled with proton pump inhibitors. Doctors will adopt appropriate treatment to the significant bleeding once occur.

3.2 Discomfort

If you experience any discomfort during the study period, whether or not related to the study, you should immediately contact your doctor, who will judge and administer appropriate medical treatment.

4. Your potential benefits

If you participate in the study, the researchers can closely monitor your disease progression and clinical events during the trial for you. If you feel uncomfortable during the trial, you can communicate with the doctor in time. Doctors monitor the dynamics of the condition at any time, master the best treatment strategy which is beneficial to you, so that you can get long-term benefits.

5. Compensation

The drugs used in this study are all conventional therapeutic drugs that have been on the market. All the examinations and laboratory tests involved are routine examinations and tests, you should bear your own expenses. Doctors will prevent possible adverse reactions, and if an adverse event occurs in a clinical trial, a committee of medical experts will determine whether it is related to rivaroxaban/aspirin. You will still bear the costs of treatment and examinations for other diseases that you have concomitant with.

6. Withdrawal

Participation in the study is entirely up to you. You may refuse to participate in the study or withdraw from the study at any time during the study, this will not affect your relationship with your doctor, and will not affect your treatment or other benefits.

The investigators may terminate your participated trial at any time during the study in your best interest. You may also be advised to have laboratory tests or a physical examination if your doctor deems it necessary.

7. Privacy

Your medical records (case reports, laboratory tests, etc.) will be kept completely in the General Hospital of Northern Theatre Command. Your doctor will record the results of laboratory and other tests on your medical record. Researchers, ethics committees, monitor and drug regulators will be allowed access to your medical record. Your personal identity will not be disclosed in any public report on the result of this study. We will do everything within the law to protect the privacy of your personal medical information. In accordance with the requirements of medical research ethics, except for personal privacy information, the trial data will be available for public inquiry and sharing, which will be limited to web-based electronic databases to ensure that no personal privacy information will be disclosed.

8. Answer questions about the study

You may ask any questions about this study at any working time and receive answers accordingly. If it is necessary to modify the content of the informed consent with unexpected clinical effects, you or your legal representative should sign again for confirmation. If there is

any significant new information during the study that may affect your willingness to continue to participate, your doctor will inform you promptly. Your doctor will be available to contact you in the event of an emergency.

It is up to you (and your family) to decide whether to participate in the study. Before you decide to join the study, ask your doctor as many questions as possible. Thank you for reading the above material and co-operation.

Please keep this document.

The present study has been approved on paper by Ethics Committee of General Hospital of Northern Theatre Command. If you have any questions about you rights in the study, please contact: Ethics Committee of General Hospital of Northern Theatre Command, No.83 Wenhua Road, Shenyang, Liaoning Province, 110016, Tel:+86-24-28856577

Informed consent (for signature)

I have read all the words of the above guidelines.

I voluntarily agree to participate in the study ”A randomized, controlled study of rivaroxaban in patients with abdominal aortic aneurysm and high-sensitivity C-reactive protein elevation (BANBOO)”

Statement of Consent

1. I have recovered Informed consent with signature and date. I understand the purpose, nature, protocol, potential risk, and benefit of this study, and other standard treatments for my disease. I have had plenty of time to ask any questions. I have been provided satisfactory answers.

2. I agree to cooperate with the research doctor and comply with the requirements of this study.

3. I have been told that I must contact with doctor if illness or symptom arises so I may receive for effective treatment.

4. I understand that this study fully follows the declaration of Helsinki, and Chinese Clinical research regulations and specifications. The present study has been approved on paper by the Ethics Committee of General Hospital of Northern Theatre Command.

5. I can choose not to participate in the study or withdraw at any time during the study. My decision will not affect my medical service.

6. My participating in the present study (even if I withdraw) means you agree with no limitation of dealing with my data in clinical research. My privacy will be kept secret and will not appear

in any research documents, reports or published articles.

I agree to participate in the study.

Subject Name (signature): Date:

Tel:

I have detailed introduced the purpose, nature, protocol, potential risks and benefits of this study. The subject has agreed to be enrolled in the study with signature and date.

Investigator (signature): Date:

Tel:

Appendix D. Ethics document.

中国人民解放军北部战区总医院

医学伦理委员分会临床试验审查批件

伦审 Y（2021）090 号

试验项目名称	利代沙班和阿司匹林应用于超敏 C 反应蛋白升高的小腹主动脉瘤患者的有效性与安全性随机对照研究		
试验产品名称	不适用	注册分类	不适用
NMPA 批件号	不适用	临床研究分类	科研
申办者/实施者	中国人民解放军北部战区总医院		
临床试验专业/科室	心内科	本院主要研究者	王效增
审查会议地点	北部战区总医院机构会议室	审查会议日期	2021 年 09 月 22 日
审查方式	■会议审查	□快速审查	□紧急会议审查
送审资料	见附件		
年度/定期持续审查频率	6 个月		

根据卫生部《涉及人的生物医学研究伦理审查办法（2016）》、NMPA《药物临床试验质量管理规范（2020）》、《医疗器械临床试验质量管理规范（2016）》、WMA《赫尔辛基宣言》和 CIMOS《人体生物医学研究国际道德指南》的伦理原则，经本伦理委员会审查，同意按所批准的临床研究方案、知情同意书、招募材料开展本项研究。

请遵循 GCP 原则、遵循伦理委员会批准的方案开展临床研究，保护受试者的健康与权利。

研究过程中，若变更主要研究者，对临床研究方案、知情同意书、招募材料等做出任何修改，请申请人提交修正案审查申请。

发生严重不良事件，请申请人按规定时限及时提交严重不良事件报告。

请按照伦理委员会规定的年度/定期持续审查频率，在截止日期前 1 个月提交研究进展报告。当出现任何可能显著影响试验进行或增加受试者危险的情况时，请及时提交书面报告。

研究出现违背方案开展研究的情况；或可能对受试者的权益/健康以及研究的科学性造成不良影响等违背 GCP 原则的情况，请提交违背方案报告。

申请人暂停或提前终止临床研究，请及时提交暂停/终止研究报告。

完成临床研究，请申请人提交结题报告。

本批件有效期为 3 年，一年内未实施的，则自行废止，需重新递交伦理审查。

主任委员（签名）：

日 期：2021 年 09 月 22 日

地址：沈阳市沈河区文化路 83 号邮编：110016 电话/传真：024-28856577
